# Plastome evolution in the East Asian lobelias (Lobelioideae) using phylogenomic and comparative analyses

**DOI:** 10.3389/fpls.2023.1144406

**Published:** 2023-03-31

**Authors:** Chun-Jiao Li, Xin-Tong Xie, Hong-Xin Liu, Ruo-Nan Wang, De-Zhu Li

**Affiliations:** ^1^College of Life Science, Shenyang Normal University, Shenyang, Liaoning, China; ^2^Germplasm Bank of Wild Species, Kunming Institute of Botany, Chinese Academy of Sciences, Kunming, Yunnan, China; ^3^School of Life Sciences, Sun Yat-Sen University, Guangzhou, China

**Keywords:** East Asian lobelias, plastomes, phylogenomic framework, highly divergent regions, positive selection, adaptive evolution

## Abstract

*Lobelia* species, as rich source of the alkaloid lobeline which has been shown to have important biological activity, have been used in folk medicine throughout East Asia to treat various diseases. However, *Lobelia* is a complex and varied genus in East Asia and is thus difficult to identify. Genomic resources would aid identification, however the availability of such information is poor, preventing a clear understanding of their evolutionary history from being established. To close this gap in the available genomic data, in this study, 17 plastomes of East Asian lobelias were newly sequenced and assembled. Although the plastomes of *Lobelia* sect. *Hypsela*, *L.* sect. *Speirema*, and *L.* sect. *Rhynchopetalum* shared the gene structure, the inverted repeat (IR)/large single copy (LSC) boundaries, genome size, and the number of repeats were variable, indicating the non-conservative nature of plastome evolution within these sections. However, the genomes of the *Lobelia* sect. *Delostemon* and *L.* sect. *Stenotium* showed rearrangements, revealing that these two sections might have undergone different evolutionary histories. We assessed nine hotspot genes and 27-51 simple sequence repeat motifs, which will also serve as valuable DNA barcode regions in future population genetics studies and for the delineation of plant species. Our phylogenetic analysis resolved the evolutionary positions of the five sections in agreement with previous evolutionary trees based on morphological features. Although phylogenetic reconstruction of Lobelioideae based on the *rpoC2* gene has rarely been performed, our results indicated that it contains a considerable amount of phylogenetic information and offers great promise for further phylogenetic analysis of Lobelioideae. Our site-specific model identified 173 sites under highly positive selections. The branch-site model exhibited 11 positive selection sites involving four genes in the East Asian branches. These four genes may play critical roles in the adaptation of East Asian taxa to diverse environments. Our study is the first to detect plastome organization, phylogenetic utility, and signatures of positive selection in the plastomes of East Asian lobelias, which will help to further advance taxonomic and evolutionary studies and the utilization of medicinal plant resources.

## 1 Introduction

Valuable medicines with anti-inflammatory, antioxidative, antiviral, and antiepileptic functions can be made from lobeline and its derivatives (Tamboli et al., 2012; Thawabteh et al., 2019). Lobeline is predominantly derived from plants of the *Lobelia* (Lammers, 2007b; Zheng et al., 2021). *Lobelia* L. is the largest genus among all the 29 genera in the subfamily Lobelioideae (Campanulaceae) and has an almost cosmopolitan distribution mainly in tropical to warm-temperate climate regions (Lammers, 2007a; Lammers, 2011). Approximately 12% of *Lobelia* species are found in Asia, with 27 of them being found in East Asia alone (Lammers, 2007a; Hong et al., 2011). East Asian lobelias are widely distributed in subtropical areas, with *Lobelia sessilifolia* extending north into eastern Siberia (Lammers, 2011). The lobelias, which have colonized East Asia, are classified into five sections based on morphological traits: *Lobelia* sect. *Delostemon*, *L.* sect. *Stenotium*, *L.* sect. *Hypsela*, *L.* sect. *Speirema*, and *L.* sect. *Rhynchopetalum* (Hong et al., 2011; Lammers, 2011).

In East Asia, *Lobelia* is a complex and varied genus, making it challenging to delineate because species and infra-specific taxa exhibit many morphological similarities. The distinctive features are confined to a few morphological traits, which are frequently ineffective for species identification, such as minor differences in plant size, plant color, and indumentum type (Li et al., 2017). For example, *L. erectiuscula* is distinct from *L. davidii* due to differences of corolla size between purple-blue corollas (1.3–1.9 cm long) and purple-red or red-purple corollas (1.1–2.8 cm long) (Li et al., 2017). Furthermore, the investigation of the herbarium field specimens revealed several intermediates and various forms. Macroscopic morphological similarity led to a high degree of misidentification between closely related taxa. Our previous study demonstrated that the leaf epidermal characteristics helped differentiate species of *Lobelia* (Li et al., 2017). However, no molecular characterization or identification research has been conducted by this group.

While many botanists have attempted to understand the phylogenetic relationships of lobelias worldwide (Antonelli, 2008; Lagomarsino et al., 2014; Knox and Li, 2017), East Asian lobelias are much less well-studied. Only three sections of East Asian lobelias have been sequenced, with genomic coverage being much lower for the *L.* sect. *Delostemon* and *L.* sect. *Stenotium* (Antonelli, 2008; Antonelli et al., 2010; Knox, 2014; Kagame et al., 2021). Previous phylogenetic studies have failed to reconstruct a well-supported phylogenetic tree forming the backbone of the East Asian clades. Many discordances between the phylogenetic relationships of East Asian lobelias inferred from nuclear ribosomal ITS (nrITS) and those inferred from a small number of plastid gene regions have been noted (Chen et al., 2016; Kagame et al., 2021). Plastid markers have been widely used for reconstructing phylogenetic relationships of plants because of their high copy numbers, conserved structure, and uniparental inheritance (Wicke et al., 2011; Gitzendanner et al., 2018). Nevertheless, the utilization of only a few plastid genes is often inadequate to determine the general or species-level phylogeny because of the low mutation rate (Hernandez-Leon et al., 2013; Shaw et al., 2014; Zhou et al., 2019). The plastid genome can be easily obtained cost-effectively using next-generation sequencing technologies, which have been successfully and extensively applied to resolve complex phylogenetic problems from deep to the shallow taxonomic levels of angiosperms (Heckenhauer et al., 2019; Thode et al., 2020). Knox and Li (2017), based on the plastid genomes of several East Asian lobelias, inferred ancestral lineages of the Hawaiian and African giant lobelias, which existed in both South Africa and Eastern Asia but subsequently underwent extinction. However, the possibility remains that they might not be sampled. Therefore, the plastid phylogenomic approach, as opposed to the previous marker-based approach, may be a better choice for elucidating the phylogenetic relationships of East Asian lobelias. Moreover, expanding the collection of fully-sequenced East Asian lobelias will allow the origin of giant lobelias to be reassessed.

Angiosperm plastid genomes are generally highly conserved in their organization, gene order, and content. However, plastid genomes from Lobelioideae are unique due to their unusual structural characteristics (Knox, 2014), showing unprecedented variations in content and gene order (Knox, 2014; Knox and Li, 2017; Li et al., 2020). Lobelioideae plastid genomes generally contain dozens of large open reading frames (ORFs, putative protein-coding genes), an uncommon feature in other angiosperms (Knox, 2014). Previous studies have also revealed extensive genome rearrangements and gene/intron loss throughout the subfamily, as well as severe reduction of inverted repeats in the genera of *Downingia* and *Isotoma* (Knox and Li, 2017). This diversity makes the plastomes of Lobelioideae an excellent basis for studying genomic evolution.

In this study, we sequenced and assembled 17 plastomes from 15 species covering all five sections of East Asian lobelias. We evaluated the sequences using a comparative genomics approach to investigate structural variation in the plastomes of East Asian lobelias. We reconstructed a phylogenomic tree to detect the phylogenetic position of East Asian *Lobelia* species within Lobelioideae and examined highly divergent regions as genetic markers. We also tested how informative the plastid protein-coding genes (PCGs) were in resolving the phylogenetic relationships between the Lobelioideae genera and sections. The nucleotide substitution rates in plastid genomes were also explored to reveal mechanisms related to the adaptation of Asian Lobelioideae species to diverse environments, especially alpine environments. This study is the first survey of plastome organization, phylogenetic utility, and the signatures of positive selection in plastids of the genus *Lobelia*, with the findings being of medicinal importance in East Asia.

## 2 Materials and methods

### 2.1 Sampling, sequencing, and assembly

A total of 82 Lobelioideae plastomes, representing 23 genera and 13 sections within the genera *Lobelia*, were obtained from previous studies (Lammers, 2007a; Knox, 2014; Crowl et al., 2016; Knox and Li, 2017). Seventeen plastomes of East Asian *Lobelia* species were newly sequenced and assembled. *Trachelium caeruleum* (Campanuloideae) and *Cyhia elata* var. *gerrardii* (Cyphioideae), two representatives of the closely related subfamilies of Lobelioideae, were selected as the outgroups. **Table S1** provides the GenBank accession numbers of all taxa used in the present study and information about newly sequenced species. Total genomic DNA was isolated from leaves of herbarium specimens using the CTAB method (Doyle, 1987). Voucher specimens were deposited in the Herbarium of the Kunming Institute of Botany, Chinese Academy of Sciences (KUN). We used agarose gel electrophoresis with Thermo Scientific NanoDrop Products to estimate the DNA quality. Total DNA was randomly fragmented into 400-600 bp fragments using an ultrasonicator, according to the Illumina Manufacturer Manual. The NEBNext^®^ Ultra™ II DNA Library Prep Kit for Illumina was used to establish the DNA libraries of 500 bp. The Illumina paired-end sequencing (2×150 bp) was performed on the Illumina HiSeq platform at the Germplasm Bank of Wild Species in Southwest China (KUN).

### 2.2 Plastome assembly and annotation

The raw reads were filtered, and the clean reads were assembled into a circular plastome using GetOrganelle (Jin et al., 2020), with a complete plastome from *Lobelia chinensis* (NC_035370) as the reference sequence. We used Bandage software (Wick et al., 2015) to view and examine the final assembly graph of the plastomes. We used Sequencher 5.4.6 (Gene Codes Corporation) to identify the junctions between the inverted repeats (IRs) and large single-copy regions (LSCs). A Plastid Genome Annotator (PGA) (Qu et al., 2019) was used to automatically annotate the plastid genome. We utilized Geneious 8.0.2 (Kearse et al., 2012) to adjust the final plastome assembly. The OGDRAW tool (Lohse et al., 2013) was used to draw a circular plastid map using default settings. We investigated the ORF(>400 bp) of the large single-copy region of each East Asian taxon using Geneious software.

Four junctions between the IRs and the small single copy (SSC)/LSC regions were validated using the sequences obtained from the Sanger sequencing of polymerase chain reaction (PCR) products. To amplify these junctions using PCR, we developed primers based on a reference sequence (*L. chinensis*) through the website application of Primer3 (http://frodo.wi.mit.edu/primer3/) by using standard options. **Table S2** shows the PCR procedure. Sanger sequencing of PCR products was performed by the Shanghai Biosune Biotechnology Company. We used Geneious 8.0.2 (Kearse et al., 2012) to align the sequences to check for significant differences.

### 2.3 Phylogenetic analyses

Seventy-four protein-coding genes were extracted from 82 Lobelioideae plastomes and outgroups and aligned individually using MAFFT (Katoh and Standley, 2014) with default options. Alignments were then adjusted manually in Geneious. We excluded *ycf1* and *ycf2* because the uncertain homology assessment could generate potentially spurious and misleading results (Braukmann et al., 2017). The absence of genes and gaps was treated as missing or ambiguous data. The optimal partitioning strategy and evolution models were selected using PartitionFinder (Lanfear et al., 2012) for phylogenetic reconstruction. Maximum likelihood (ML) trees were reconstructed with RAxML using a standard bootstrapping (BS) approach with 1000 replicates (Stamatakis, 2006) under the GTR+I+G model, which was selected by PartitionFinder as the optimal model (**Table S4**). We employed the JModelTest 2.0 program (Darriba et al., 2012) to ascertain the most appropriate model dataset given the Akaike Information Criterion. In addition, the Bayesian inference (BI) tree was reconstructed in MrBayes version 3.2 on the concatenated genes and partitioned based on the best model as determined with Partition Finder. The Markov chain Monte Carlo (MCMC) analysis was performed over 100 million steps and was completed every 10,000 generations. We discarded the first ten million of all the sampled trees as burn-in and determined the consensus tree from the remnant trees. Effective sample size (ESS) values (>200) were used to examine stationarity using Tracer 1.6 (Rambaut et al., 2014). We used the Figtree v.1.4.0 program (Rambaut, 2012) to visualize and annotate trees.

### 2.4 Comparative plastome analysis

We excluded samples of *Lobelia alsinoides* and *L. fangiana* without the complete plastomes as well as repeated species of *L. zeylanica*_S11412. The length of the total plastome, the coding region, the non-coding region, and the GC content were calculated using Geneious 8.0.2. To observe the potential existence of IR contraction or expansion among all Asian lobelia plastomes, we used *L. chinensis* as a reference. We detected the IR/LSC and IR/SSC borders using the online software IRScope (Amiryousefifi et al., 2018). The percentage of sequence identity was plotted using the mVista program in shuffle-LAGAN mode (Brudno et al., 2003; Frazer et al., 2004). The IR region from each plastome was removed to check for repeats. REPuter tool (Kurtz et al., 2001) was used to investigate the dispersed repeats in each plastome, with a minimum repeat size of 20bp and a Hamming distance of 3. Repeats were categorized into three classes based on length (20-50 bp, 50-100 bp, and more than 100 bp), then visualized in R v.3.5.1 (R Core Team, 2020).

### 2.5 Identification of DNA markers

We examined SSRs throughout the genomes of 18 Asian lobelias using the Perl script MISA (Thiel et al., 2003). The minimum thresholds were 12, 6, 5, 5, 5, and 5 for the corresponding mono-, di-, tri-, tetra-, penta-, and hexanucleotide repeats, respectively. Because there were many structural rearrangements among the studied Lobelioideae plastomes, we compared nucleotide sequence divergences across the 74 plastid coding genes only to identify the plastid genome hotspots. Newly assembled and publicly available plastomes from Lobelioideae, including one with an inverted repeat region, were aligned using the MAFFT multiple sequence alignment program (Katoh and Standley, 2014). Nucleotide diversity (pi) throughout the plastid protein-coding regions was detected using the DNASP software package V.6.11.01 (Librado and Rozas, 2009). The window size was 600 bp, and the stepping size was 200 bp.

### 2.6 Phylogenetic informativeness for protein-coding genes

The PhyDesign online application was implemented with the standard settings (López-Giráldez and Townsend, 2011) to profile the phylogenetic informativeness of the 74 plastid coding sequences. We used the maximum likelihood reconstruction of concatenated coding genes. The ultrametric tree given in relative time was converted using the dnamlk program from Phylip v.3.2 (Felsenstein, 1989). Our input files in PhyDesign were the aligned 74 genes and the relative-time ultrametric tree.

### 2.7 Genes and sites under the positive selection

We excluded the *ndh* genes, *rpl23*, and *infA* from this analysis because some species had lost these sequences. To determine the PCG sequences affected by the species selection of Lobelioideae, we implemented the MUSCLE algorithm in MEGA 7.0 (Kumar et al., 2016) to align each gene. The ML phylogeny deduced from RAxML was employed as the constraint topology. First, we implemented the site-specific evolutionary selection, including M0, M1a, M2a, M3, M7, and M8 in PAML3.15 software (Yang, 2007). This model assumes that the ω ratio varies among sites but that all lineages share a single ω ratio. The following comparisons were performed: M0 (one-ratio)/M3 (discrete), M1a (nearly neutral)/M2a (positive selection), and M7 (β)/M8 (β and ω). The likelihood ratio test (LRT) was used to assess the strength of the selection. *P* values from chi-squared tests (x^2^) (<0.05) were regarded as significant.

To detect the influence of positive selection on the sites of the foreground branch, we constructed the branch-site model of the CodeML program in EasycodeML v.1.31 (Gao et al., 2019). This model allowed the ω ratio to vary among foreground lineages and within phylogenetic sites. The five sections were treated separately as foreground branches. We could also individually detect regions of positive selection in the above lineages using 63 PCGs. In cases where *p*-values were reported as significant (<0.05), we calculated posterior probabilities of positively selected sites on these lineages (Dhar et al., 2020) using the Bayes empirical Bayes (BEB) approach.

## 3 Results

### 3.1 Plastomes characteristics

**Figure 1:**
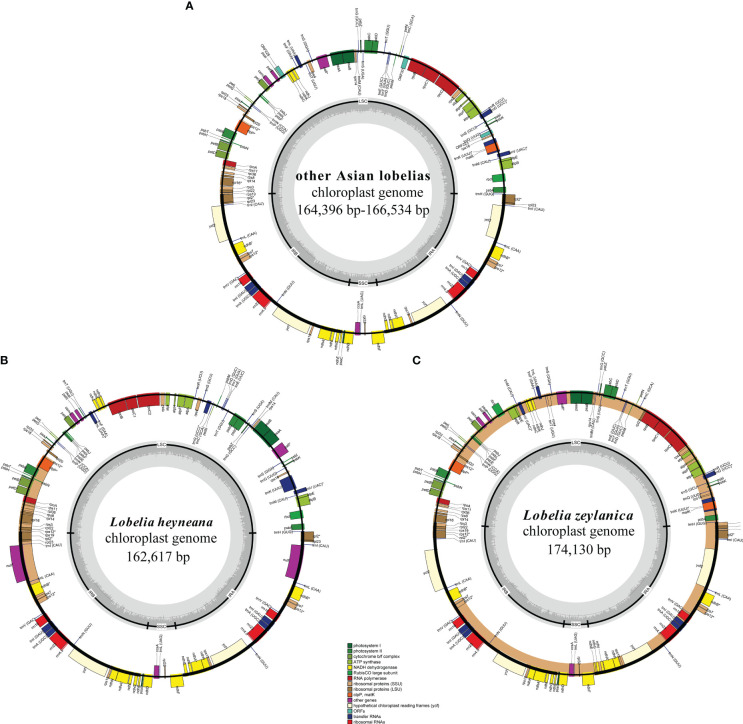
Plastid genomes of other East Asian lobelias **(A)**, *Lobelia heyneana* **(B)**, and *Lobelia zeylanica* **(C)**. Genes inside the circle are transcribed clockwise, and genes outside the circle are transcribed counter-clockwise. The dark-gray inner circle corresponds to the GC content, and the light-gray represents the AT content.

Clean reads of the newly assembled genomes ranged in number from 12,024,606 to 20,781,623. Fourteen plastomes were complete, not including *L. alsinoides*, *L. zeylanica*_S11412, and *L. fangiana* since they could not be determined as circular, despite all the protein-coding genes being identified. All complete plastomes exhibited a typical quadripartite architecture comprising an LSC region, an SSC region, and two IRs. Their lengths ranged from 162,617 bp in *L. heyneana* to 174,130 bp in *L. zeylanica* (**Table 1**). Eleven protein-coding genes and seven tRNA genes were interrupted by introns, three of which contained two introns. Three trans-spliced exons were predicted from the *rps12* gene. Plastomes shared the same gene order except for *L. zeylanica* and *L. heyneana*, which showed gene order changes in the LSC region (**Figure 1**). Species of *L.* sect. *Hypsela*, *L.* sect. *Speirema*, and *L.* sect. *Rhynchopetalum* had three typical ORFs (>400 bp) occurring in *rps16-trnQ(UUG)*, *ndhC-psaI*, and *rpoB-trnC(GCA)* in the LSC regions (**Table S3**). However, *L. zeylanica* and *L. heyneana* did not exhibit these ORFs in the same regions. The largest number of dispersed repeats was observed in *L. zeylanica* and *Pratia angulata*, followed by *L. melliana*. Most accessions contained fewer than 300 dispersed repeats (**Appendix S1**).

**Table 1:** Assembly information of twenty plastomes of East Asian lobelias.

Section	Species	Genome Size (bp)	LSC (bp)	SSC (bp)	IR (bp)	Intergenic sequences (bp)	Number of genes	Protein-coding genes	tRNA	rRNA	Total GC content(%)	Protein-coding GC(%)
*L.* sect. *Delostemon*	*L. zeylanica*_S03968	174,130	82,611	7,341	42,089	83,035	134	91	35	8	38.3	38.5
*L.* sect. *Stenotium*	*L. heyneana*	162,617	79,831	7,884	37,451	74,102	130	87	35	8	39.1	39.1
*L.* sect. *Hypsela*	*L. chinensis*	164,396	82,460	7,962	36,987	75,074	131	87	36	8	39.0	38.9
	*Pratia angulata*	164,856	82,620	7,960	37,138	75,708	131	87	36	8	39.0	38.9
	*P. nummularia*_S08798	165,496	83,005	7,991	37,250	76,480	130	86	36	8	39.0	38.9
	*P. nummularia*	165,428	82,764	7,982	37,341	75,539	131	87	36	8	39.0	38.9
*L.* sect. *Speirema*	*L. montana*	166,239	83,841	7,970	37,214	78,552	127	84	35	8	39.1	38.9
*L.* sect. *Rhynchopetalum*	*L. sessilifolia*	165,656	83,217	7,987	37,226	76,691	132	87	37	8	39.1	38.9
	*L. melliana*	165,413	82,611	7,618	37,592	75,278	132	87	37	8	39.0	38.9
	*L. pyramidalis*_S11387	165,024	82,881	7,919	37,112	75,891	126	84	34	8	39.0	39.1
	*L. pyramidalis*_S13267	165,843	83,436	7,967	37,220	77,238	126	84	34	8	39.1	39.1
	*L. seguinii*	166,534	84,002	7,960	37,286	77,428	131	87	36	8	39.0	39.0
	*L. davidii*	166,299	83,711	7,962	37,313	77,493	125	86	31	8	39.1	39.1
	*L. colorata*	166,173	83,659	7,960	37,277	79,014	123	84	31	8	39.0	39.1
	*L. erectiuscula*	165,119	83,013	7,976	37,065	77,549	126	86	32	8	39.0	39.2
	*L. taliensis*	164,928	82,993	7,977	36,979	78,339	124	84	32	8	39.0	39.2
	*L. pleotricha*	164,966	82,904	7,978	37,042	78,479	124	84	32	8	39.0	39.2
	*L. doniana*	165,066	83,181	7,975	36,955	78,225	124	85	31	8	39.0	39.2
	*L. iteophylla*	165,228	83,116	7,962	37,075	77,463	126	86	32	8	39.0	39.2
	*L. clavata*	165,295	83,092	7,981	37,111	77,176	125	86	31	8	39.0	39.2

**Figure 2:**
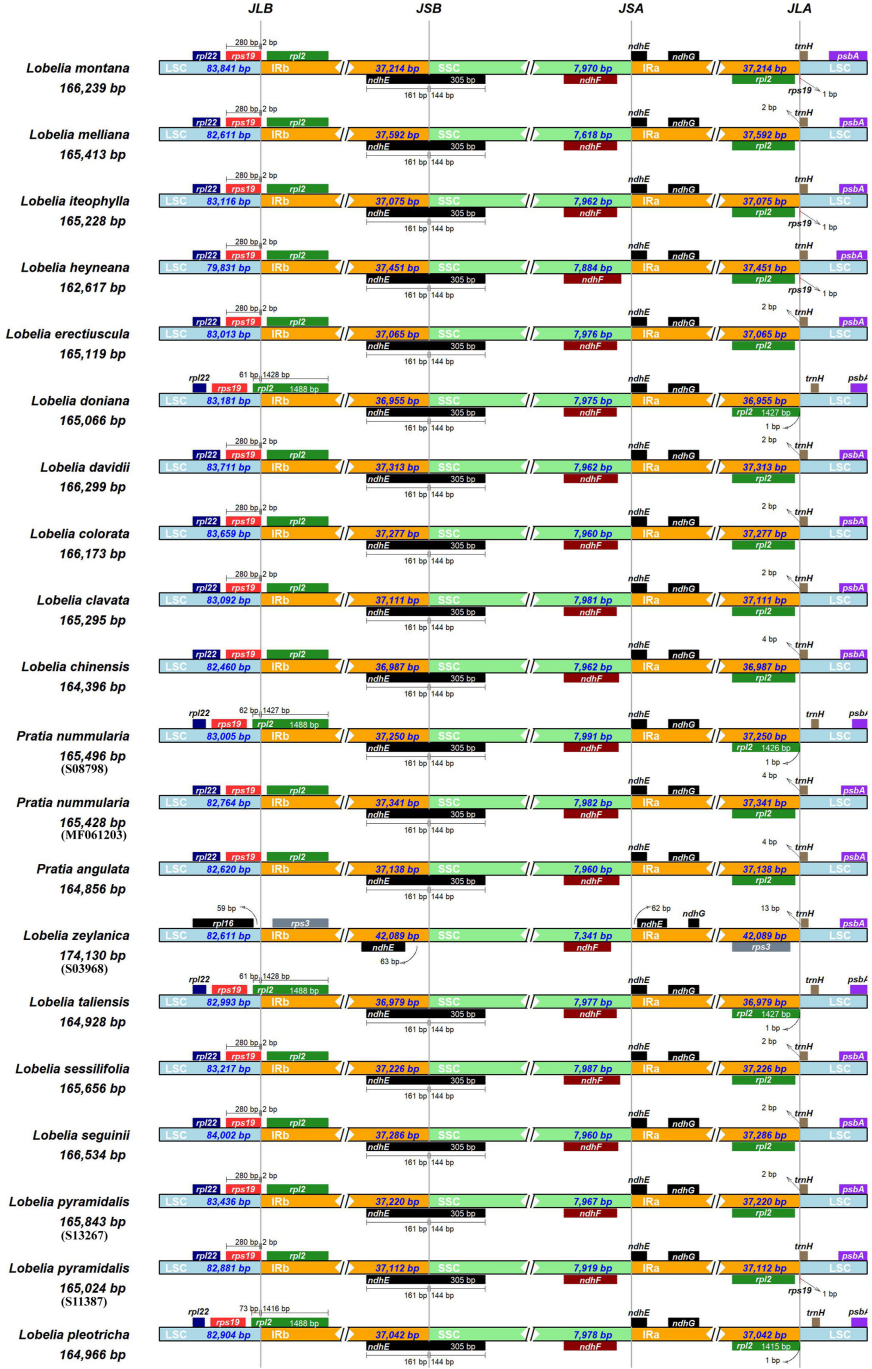
Comparison of IR-SC border positions across plastomes of twenty East Asian lobelias. Genes are denoted by colored boxes. The gaps between the genes and the boundaries are indicated by the base lengths (bp).

The pattern of IR regions was not highly consistent among the plastomes of Asian lobelias with a length of 36,955-42,089 bp. The intergenic region between *rpl16* and *rps3* was inserted at the IRb/LSC junction in the *L. zeylanica* plastome. This junction was located within the *rpl2* gene of *L. heyneana*, *L. doniana*, *L. taliensis*, and *L. pleotricha* plastomes. The IRb/LSC junctions in all other species were nested within the *rps19* gene. The junctions of the IRa/SSC regions in most accessions were situated in the *ndhE* gene, although it laid between *ndhF* and *ndhE* in *L. zeylanica* plastome. The *rpl2* and *rps19* genes were found at the IRb/LSC junction of *Pratia nummularia*_S087983 and *P. nummularia*_MF061203, respectively (**Figure 2**).

**Figure 3:**
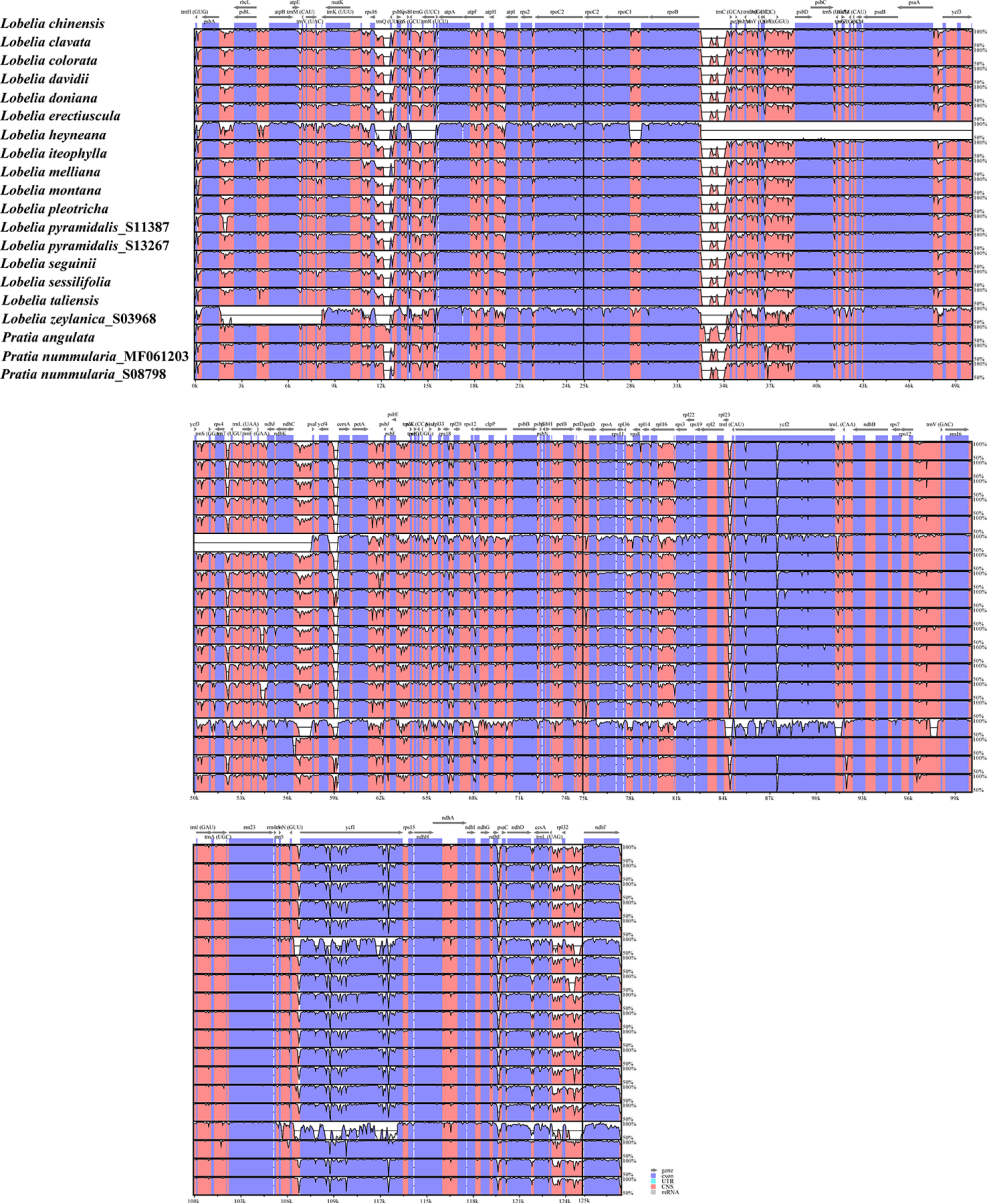
Visualization of the alignments of twenty plastomes representing all sections of East Asian lobelias using mVISTA, with *Lobelia chinensis* as the reference sequence. The gray arrows above the alignment indicate genes. Different colors represent different regions (coding and non-coding). The horizontal axis indicates the coordinates within the plastid genome. The vertical scale represents the percentage of identity, ranging from 50% to 100%.

To explore the sequence divergence of the plastome regions, we implemented the mVISTA program to plot the percentages of sequence identities, with *L. chinensis* as a reference. In addition to the plastomes of *L. zeylanica* and *L. heyneana*, the high similarity was found among other accessions, revealing that these plastome sequences were conserved. The IRs were more conserved than the LSC and SSC regions (**Figure 3**). Divergences were observed in both coding regions and non-coding regions. Coding regions, such as *ycf1*, *ycf2*, *rpoC2*, *ccsA*, *ndhD*, and *ndhF*, were frequently non-conserved. Seven non-coding regions showed substantial divergence in all intergenic spacers: *rps16*-*trnQ(UUG)*, *rpoB*-*trnC(GCA)*, *ndhC*-*psaI*, *petA-psbJ*, *psbE-petL*, *trnL(UAG)-rpl32*, *rpl32-ndhF* (**Figure 3**). The *L. heyneana* plastome possessed the ~25, ~1.5, and ~0.8 kb inversions, and the most significant inversion was located between *ropB* and *psaI*. *L. zeylanica* had a ~6 kb inversion between the *psbA* and *matK*.

### 3.2 Molecular markers

**Figure 4:**
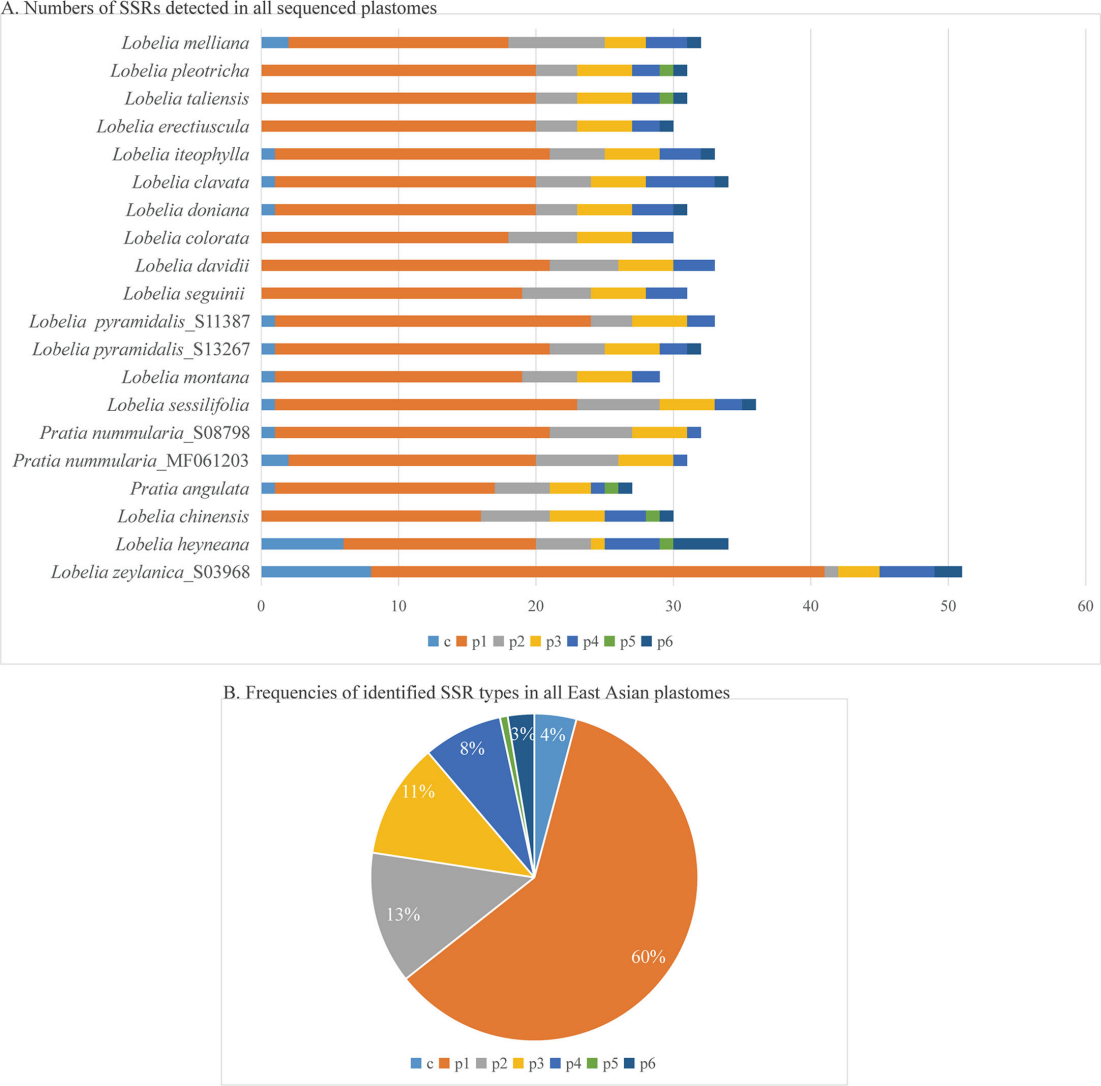
Comparison of simple sequence repeats (SSRs) among Asian plastomes. **(A)** Numbers of SSRs detected in all sequenced plastomes. **(B)** Frequencies of identified SSR types in all plastomes of East Asian species. p1, mononucleotides (mono-); p2, dinucleotides (di-); p3, trinucleotides (tri-); p4, tetranucleotides (tetra-); p5, pentanucleotides (penta-); p6, hexanucleotides (hex-); c, compound.

**Figure 5:**
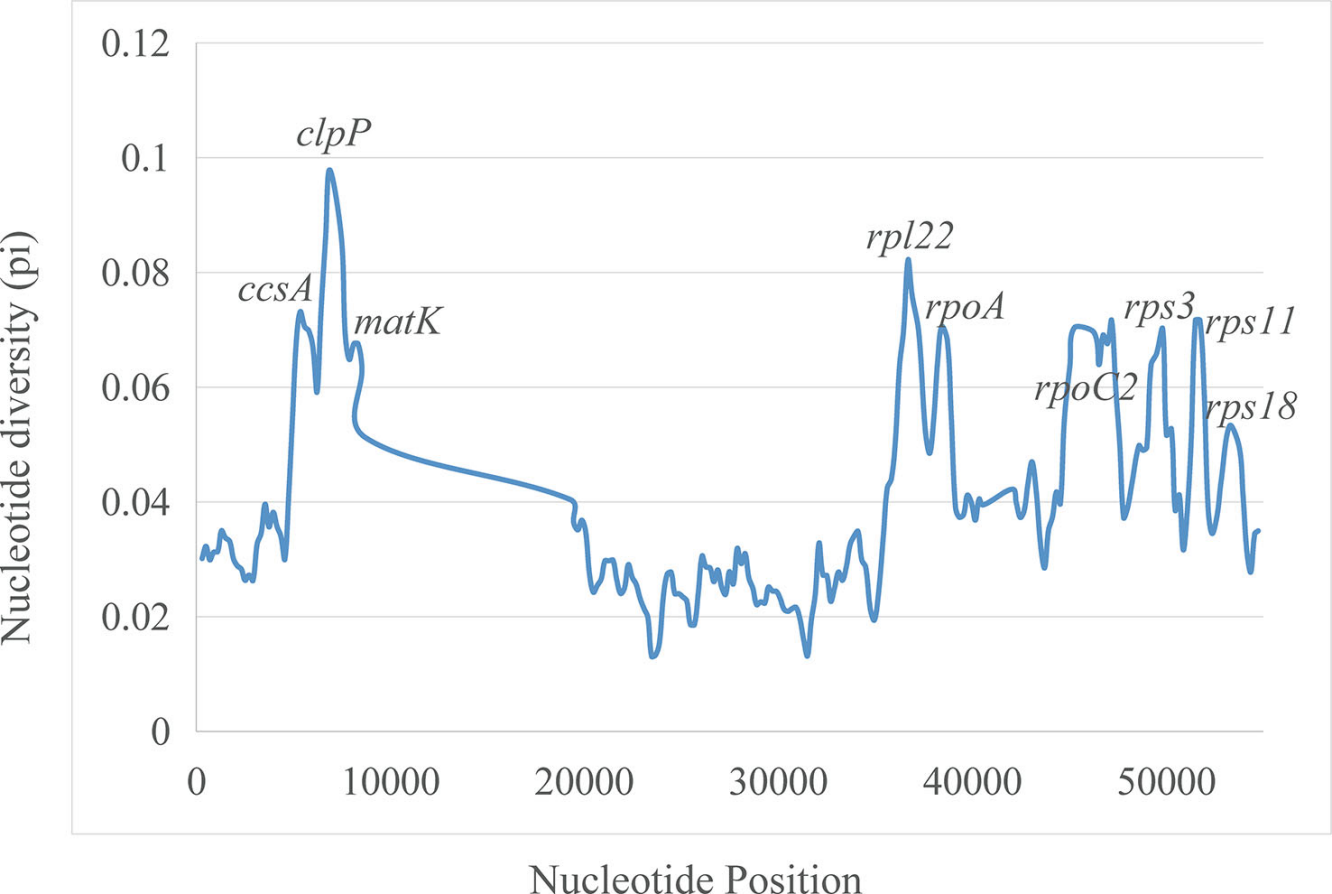
Sliding window analysis of nucleotide diversity (pi) along the twenty aligned plastomes of East Asian lobelias with one IR copy included. Genes underlying peaks of nucleotide diversity are labeled.

The plastomes showed similar SSRs counts (**Figure 4**). However, *L. zeylanica*, *L. heyneana*, and *L. sessilifolia* had higher SSRs than the other studied accessions. The number of simple sequence repeats ranged from 27 (*P. angulata*) to 51 (*L. zeylanic*a) (**Figure 4A**). A frequency of 60% was calculated for the mononucleotide repeat patterns. Dinucleotide and trinucleotide repeats had a frequency of more than 10% (**Figure 4B**). The frequencies of compound, tetranucleotide, pentanucleotide, and hexanucleotide repeats were less than 10%. For sliding window analyses (**Figure 5**), we computed a mean value of 0.03853 (pi) for the Campanulaceae species. We also observed some highly divergent regions, namely *ccsA*, *clpP*, *matK*, *rpl22*, *rpoA*, *rpoC2*, *rps3*, *rps11*, and *rps18*, in the SSC and LSC regions.

### 3.3 Phylogenetic relationships

**Figure 6:**
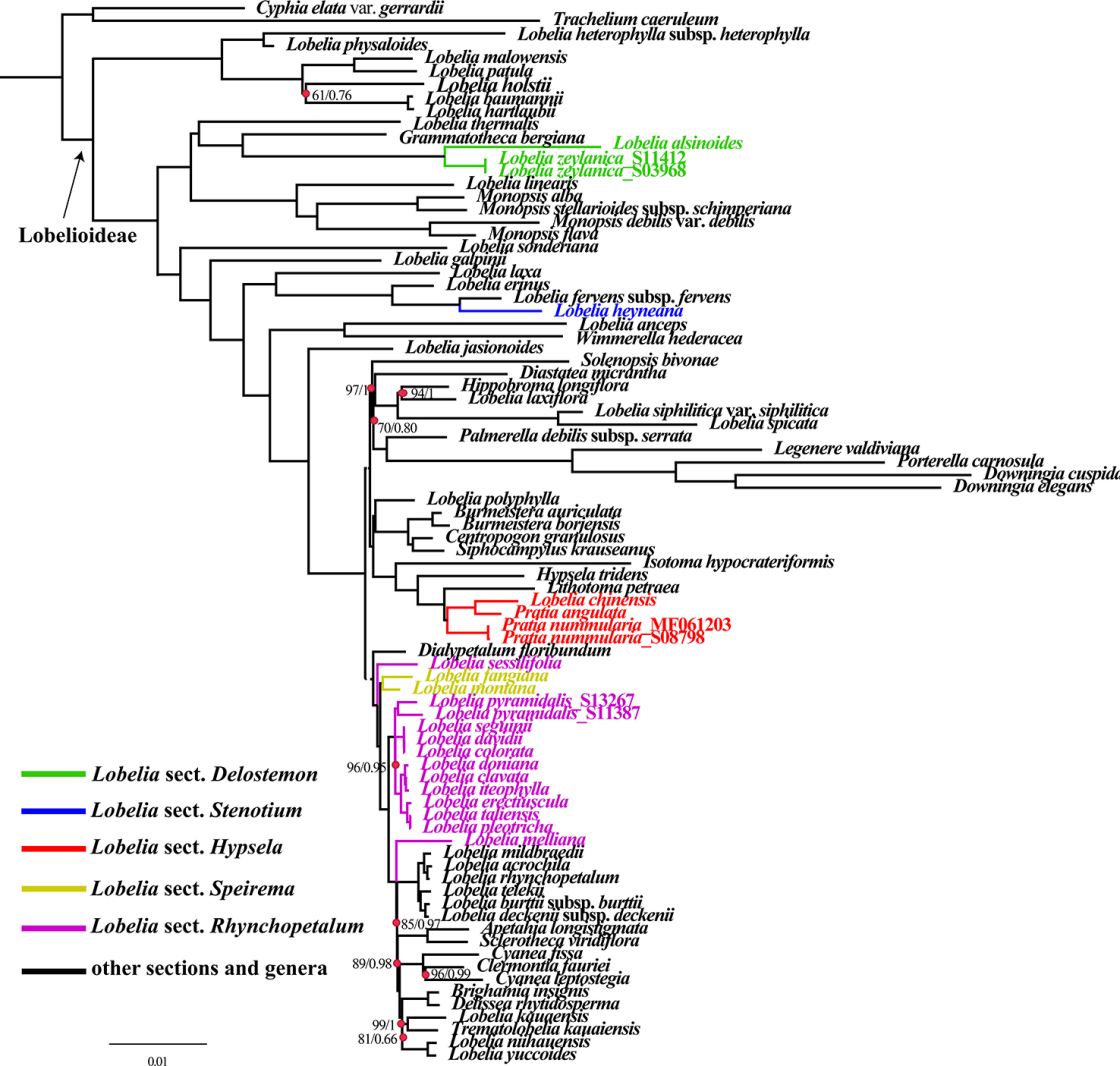
Phylogenetic relationships of all Lobelioideae species using the 74 protein-coding genes, based on the analyses of Maximum likelihood (ML) and Bayesian inference (BI).

The obtained alignment of the 74 plastid PCGs from the 84-species data set comprised 55,000 bp with 14,492 parsimony-informative sites. Partition Finder detected 22 subsets, and the best-fit partitioning schemes are described in **Table S4**. ML and BI nucleic acid analyses using partitioning schemes yielded identical tree topologies based on the 74 aligned protein-coding genes shared by all sampled species (**Figure 6**). Plastid phylogenomics has clarified the relationships between the major lineages of Lobelioideae. Bootstrap values (less than 100%) and posterior probabilities (PP<1.0) are labeled in the maximum likelihood tree.

Phylogenetic analyses revealed that the genus *Lobelia* was polyphyletic. *Lobelia* sect. *Delostemon* was polyphyletic because this section consisted of not only the East Asian species, namely *L. zeylanica* and *L. alsinoides* (**Figure 6**) but also *L. baumannii* and *L. holstii*e. *L.* sect. *Stenotium*, including *L. galpinii*, *L. anceps*, and the Asian *L. heyneana*, were polyphyletic. *L.* sect. *Rhychopetalum* included the monophyletic *L.* sect. *Speirema* (*L. fangiana* and *L. montana*), indicating that this section is not monophyletic. Most of the included sections were polyphyletic. *L.* sect. *Hypsela* was found to be monophyletic based on the taxa selected in our phylogenetic analyses. East Asia lobelias were scattered throughout the phylogenetic tree of Lobelioideae. *L. zeylanica*_S11412, *L. zeylanica*_S03968, and *L. alsinoides* were nested in a sister clade to *Grammatotheca bergiana*. *L. heyneana* emerged as a sister species to *L. fervens* subsp. *fervens*. *Pratia. nummularia*, *P. angulata*, and *L. chinensis* formed a sister clade with *Lithotoma petraea*. *L. sessilifolia* (collected from the Russian Federation, based on a previous study) showed a sister relationship with a lineage of many other East Asian lobelias and giant lobelias. *L. fangiana* and *L. montana* clustered as sister clades for most Asian taxa. *L. pyramidalis*_S13267 and *L. pyramidalis*_S11387 were placed as sisters to a clade that included the most of Asian lobelias with strong support (BP=96, PP=0.95). *L. melliana* was a sister to plants from East Africa, Hawaii, and the South Pacific, an evolutionary relationship that was moderately supported (BP=89, PP=0.98) (**Figure 6**).

### 3.4 Phylogenetic informativeness

**Figure 7:**
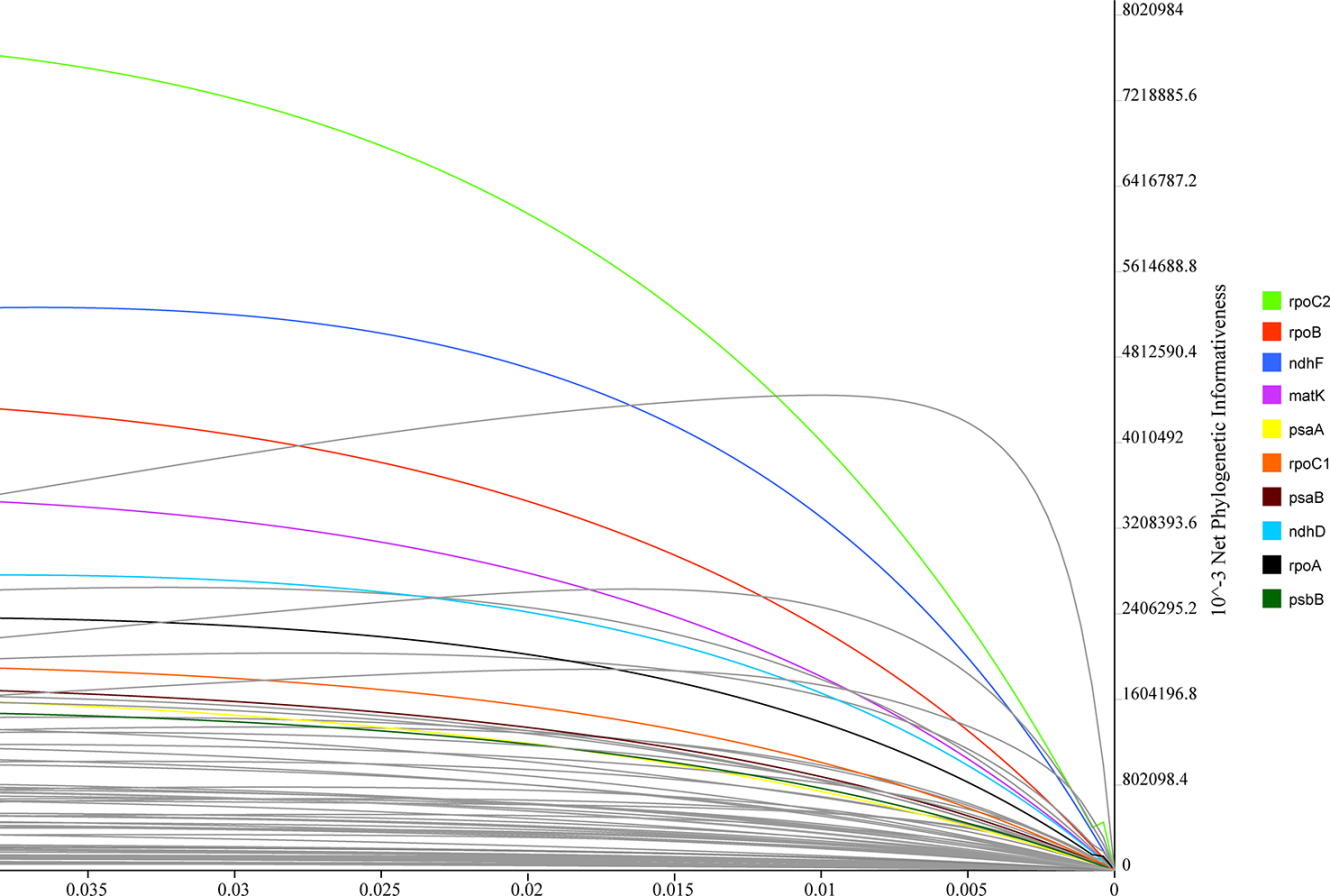
Phylogenetic net informativeness profiles of 74 protein-coding genes of Lobelioideae estimated using PhyDesign. Ten genes with the greatest informativeness are color-coded and indicated on the right. X- and Y-axes represent relative-time and net phylogenetic informativeness, respectively.

PhyDesig was used to evaluate the profiles of the per-site and net phylogenetic informativeness (PI) values of the 74 genes (**Figure 7**). The maximum net PI value was observed for *rpoC2*, followed by *rpoB*, *ndhF*, and *matK*. PCGs with high net phylogenetic informativeness values had longer lengths, suggesting that gene length might contribute significantly to phylogenetic information. *rpoC2* also performed well in the analysis of per-site PI, followed by *rpoB* and *matK* (**Table S5**). Genes with less net PI were relatively conservative and tended to be primarily related to the subunits of the ribosome or ATP synthase or shorter in length.

### 3.5 Positive selection

We used the results of BEB to determine sites under highly positive selection (P>99%,**). Ten slightly positive sites (P>95%) were also detected. Sites under positive selection within twenty-four genes were predicted (**Table S6**). The *clpP* and *rps7* genes contained the most sites under positive selection, at more than 20 sites, followed by *cemA* (2, 2), *matK* (8, 3), *rbcL* (9, 6), *rpl2* (0, 3), *rpl16* (5, 5), *rpoA* (3, 3), *rpoB* (0, 8), *rpoC1* (0, 1), *rps2* (2, 3), *rps3* (2, 3), *rps4* (2, 3), *rps8* (5, 7), *rps11* (7, 7), *rps14* (4, 4), *rps15*(1, 2), and *rps18* (2, 2). The LRT results further supported the positively selected codon sites identified (**Table S7**). A branch-site model that does not permit positive selection (Model A_null_) was compared with a model in which positive selection on the specified branches was allowed (Model A). For *L.* sect. *Stenotium* and *L.* sect. *Delostemon*, we used this model to detect 11 positive selections in five PCGs: *atpF* (two sites), *clpP* (three sites), *matK* (two sites), *psbK* (one site), and *rbcL* (three sites) (**Table 2**).

**Table 2:** Genes and sites under positive selection in the plastomes of East Asian lobelias.

Gene	Foreground-branch	LRT *p*-value	Positively selected sites
*atpF*	*L.* sect. *Stenotium*	0.032835616	40 V 0.954*, 73 G 0.541*
*clpP*	*L.* sect. *Delostemon*	0.023446531	56 S 0.969*, 66 Y 0.952*, 70 R 0.703*
*matK*	*L.* sect. *Delostemon*	0.043041741	192 N 0.921*, 282 Y 0.723*
*psbK*	*L.* sect. *Stenotium*	0.000665252	20 S 0.970*
*rbcL*	*L*. sect. *Delostemon*	0.006341115	145 V 0.530*, 320 M 0.582*, 459 C 0.558*

Sites under positive selection were marked. *represented sites under positive selection at posterior probabilities > 0.95.

## 4 Discussion

### 4.1 Plastome features

In the present study, the plastomes of all East Asian lobelias were found to have many dispersed and longer repeats and exhibited structural variations. Repeats have been reported to be associated with architectural variations in the plastid genome (Huang et al., 2014; Knox, 2014; Choi et al., 2019). Large ORF was not observed in the LSC regions of *L. zeylani*ca and *L. heyneana*. However, the other 18 accessions with large LSC regions contained three typical ORFs, suggesting that ORFs existing in the LSC regions might be relevant to the expansion of the LSC region. These ORFs have been found in some plastomes of Campanulaceae (Knox, 2014), indicating that such a genome structure may reflect their shared evolutionary history.

We demonstrated that the IRb/LSC border occurs at different positions within *rps19* or *rpl*2. In contrast, the IRa/LSC borders were located in the regions of *rpl2-trnH-GUG* for most Asian lobelias. Angiosperms shared this common IRa/LSC boundary, except for monocots whose *trnH* gene is located in the regions of *rpl2-rps19* (Wang et al., 2008; Givnish et al., 2018). The *L. zeylanica* plastid genome had a shorter LSC region (82,611 bp) but a larger IR region (42,089 bp) than the other Asian lobelias studied, which might be associated with the expansion of the junctions between the LSC and IR regions (Bendich, 2004; Li et al., 2020). *L. taliensis*, *L. doniana*, and *P. nummularia*_S08798 exhibited contractions of the IR region that had not yet been identified. A possible interpretation is that it is an illegitimate recombination event (Blazier et al., 2011; Downie and Jansen, 2015). *P. nummularia*_S08798 and *P. nummularia_*MF061203 showed different LSC/IRb junctions, which require further research in the future. Large-scale IR expansion/contraction has been illustrated to be a vital contributor to the plastome size variation (Ruhlman and Jansen, 2014; Shen et al., 2020). IR expansion/contraction is not uncommon in angiosperm plastid genomes and is known to occur in some groups, such as *Pelargonium* (Weng et al., 2017), *Passiflora* (Rabah et al., 2019), and *Euphorbia* (Wei et al., 2021).

We analyzed 20 complete plastomes of East Asian lobelias. The gene content of the plastomes was similar to that of other angiosperms. Despite the shared gene structure among the plastomes of *L.* sect. *Hypsela*, *L.* sect. *Speirema*, *L.* sect. *Rhynchopetalum*, IR/LSC boundaries, genome size, and the number of repeats were variable, indicating the non-conservative nature of plastomes evolution within these sections. *L. zeylanica* and *L. heyneana* had structural rearrangements (including large inversions) in the LSC regions that were not identified in other accessions. Therefore, this could be attributed to the different evolutionary histories of the *L.* sect. *Delostemon* and *L.* sect. *Stenotium*. Based on a comparison of plastome architectures from diverse land plant species, IR boundary shifts and inversions are the main processes of structural rearrangements, with frequency variations among lineages (Mower and Vickrey, 2018; Mower et al., 2019). Inversions have been identified in scattered angiosperm lineages; for example, the ~36 kb inversion detected in most core Genistoids (Martin et al., 2014), the ~125 kb inversion identified across the family Campanulaceae (Knox, 2014), and the ~49 kb inversion found in Circaeasteraceae (Sun et al., 2017). Large inversions are predicted to have significant value in constructing phylogenies because of their rarity, easily identified homologous features, and the deduced state polarization (Jansen et al., 2008; Shrestha et al., 2019).

### 4.2 Molecular markers

East Asian lobelias are traditional medicinal plant species because of their bioactivity and many chemical constituents (Anand et al., 2009; Han et al., 2013; Zheng et al., 2021). However, the scarcity of genomic resources for East Asian lobelias has hampered plant genetics, ecology, taxonomy, and resource utilization. The plastomes assembled in the present study provide new insights into the evolutionary history of plastids and offer valuable plant genetic resources which will be helpful in future genetic and taxonomic research of lobelia species in East Asia (Lee et al., 2019). Mononucleotide repeat and microsatellites were the most numerous markers and have also been described in other lineages such as Cyatheaceae (Zhu et al., 2021) and Campanuloideae (Li et al., 2020).

Our results indicated that the plastomes of Lobelioideae exhibited high levels of divergence in eight PCGs, which could function as useful genetic information beneficial for species authentication and phylogenetic assessment. The protein-coding region of *clpP* (pi close to 0.1) exhibited higher nucleotide diversity in all plastomes. Several studies (Kuo et al., 2011; Dugas et al., 2015; Peterson et al., 2022) have revealed different plastid genes such as *matK*, *ccsA*, and *clpP* used in the phylogenetic reconstructions at various taxonomic levels. For the herbal medicinal genus *Lobelia*, the highly variable regions acquired from this study may be promising molecular barcodes for authenticating herbarium specimens and medicinal quality assurance.

### 4.3 Phylogenetic relationships of East Asian lobelias

Twenty-nine genera constitute the Lobelioideae (Lammers, 2007a; Lammers, 2007b). Phylogenetic relationships within the broad Lobelioideae remain unclear, mainly because of the many segregates detached from *Lobelia.* This large genus has been found to be polyphyletic using molecular data (Antonelli, 2008; Knox, 2014; Knox and Li, 2017), which was supported by the synthesis of diverse phenotypic data (Lammers, 2011). *Lobelia* is one of the most complex genera across Lobelioideae and shows morphological diversity and a sub-cosmopolitan distribution (Lammers, 2011). None of the previous phylogenetic studies have sampled all five sections of the East Asian lobelias. Therefore, in this study, 17 new plastomes from all sections were sequenced and combined with previously published data to reconstruct the phylogeny. Most nodes were supported with higher BP and PP values, indicating that the relationships were robust. This phylogenomic framework confirmed the phylogenetic position of the East Asian lobelias within the Lobelioideae subfamily. The sectional partition of these species, shown in the phylogenetic tree, coincided with the classification of Lammers (2011). Our results indicated that *L.* sect. *Delostemon* was the earliest diverging group and *L.* sect. *Stenotium* was the transitional group, whereas *L.* sect. *Rhynchopetalum* was the derived group and comprised giant lobelias.

We showed for the first time that *L. heyneana* is a sister species to various African species. *L. zeylanica* and *L. alsinoides* are closely related, which is consistent with their morphological classification (Hong et al., 2011). *L. zeylanica* and *L. alsinoides* were sisters to *Grammatotheca*, which is endemic in South Africa. *L.* sect. *Hypsela* and Australasian species were in sister clades, in agreement with the results of previously published studies (Chen et al., 2016; Knox and Li, 2017). The close relationships among *L. seguinii*, *L. colorata* and *L. davidii*, were strongly supported, revealing the discordance between our study and the observations of an earlier study which positioned *L. sequinii* close to *L. melliana* (Chen et al., 2016). This inconsistency likely resulted from the sparse taxon samples and the few phylogenetic markers used. *L. pyramidalis* differs from *L. seguinii* only in corolla color and capsule shape (white or rose corolla and subglobose capsule versus purple-red or purple-blue corolla and ellipsoid capsule) (Hong et al., 2011). Although *L. seguinii* and *L. pyramidalis* are morphologically similar, they are not closely related. *L. doniana* was once considered a variant of *L. seguinii* by Wimmer (Pfl. R. Heft 107: 651. 1957) as their appearances are similar, albeit with subtle morphological differences. These are now considered separate species (Lammers, 2011), as confirmed by our phylogenetic analysis which did not find a close relationship. Although some pairwise species are morphologically similar, they may not be each other most relative lineages if two closely related taxa are present in divergent surroundings or if the lineages are undergoing adaptive evolution (Losos, 2011). The results of our study did not alter the ancestry of giant lobelias established by previous sampling studies (Chen et al., 2016; Knox and Li, 2017). The newly sequenced East Asian samples added in the present study will limit long-branch attraction in phylogenetic analysis, which should improve resolution and support at the base of the Oceania clades.

### 4.4 Phylogenetic informativeness of plastid genes

Although some advances in the analyses of phylogenetic reconstructions within Asian lobelias have been made using plastid markers, few studies have detected the sequence utility of plastomes for phylogenetic inferences in this group (Knox and Li, 2017). The severe bottleneck in the molecular phylogeny of recent diversification can be attributed to the insufficient resolution generated by the lack of phylogenetic signals (Chen et al., 2016). Therefore, identifying the plastid markers which show the highest PI values will help consolidate efforts to resolve complex phylogenies at the species level and identify plant DNA barcodes. *matK* and *ndhF* genes have been extensively used in the phylogenetic analyses of Lobelioideae (Koopman and Ayers, 2005; Antonelli, 2008). However, two longer genes (*rpoC2*, *rpoB*) with higher PI values have not yet been used in the phylogenetic reconstruction of Lobelioideae. It has been reported that the *rpoC2* gene performed well in reconstructing angiosperm phylogenies (Walker et al., 2019). Therefore, the utility of the *rpoC2* gene as a valuable plastid locus may be helpful in the phylogenetic inferences of Lobelioideae. The present study provides a standard procedure for genetic marker selection. Primer availability is crucial for a realistic assessment of the PI of markers in deep learning.

### 4.5 Signatures of positive selection on plastid genes and sites

*Lobelia* is almost cosmopolitan but is most diverse in the tropics and subtropics, with several taxa extending into temperate regions. Most East Asian lobelia species are found in high-altitude environments (Lammers, 2011). Thus these plants are continuously exposed to super ultraviolet radiation, a capricious climate, low temperatures, and high oxygen levels (Askew, 2002; Zhang et al., 2016). The site-specific model revealed 173 sites under highly positive selection in the 63 PCGs with posterior probabilities greater than 0.99. Positively selected genes have functions in both photosynthesis and the plastid genetic system, illustrating that the functional genes in the plastid genome significantly affect plant evolution (Hao et al., 2010; Jiang et al., 2018; Gao et al., 2022). In addition, positive selection is associated with shifts in function and the environment (Hu et al., 2014; Li et al., 2018). Thus, the sites under positive selection determined in this study might drive the changes in PCGs, allowing adaptation to diverse and harsh habitats (Li et al., 2018; Dhar et al., 2020; Peng et al., 2022).

We identified five genes (*atpF*, *clpP*, *matK*, *psbK*, and *rbcL*) that putatively underwent positive selection within the species of *L.* sect. *Stenotium* and *L.* sect. *Delostemon*. The *atpF* gene encodes a subunit of ATP synthase, which is essential for photosynthetic energy transduction (Walker, 2013). The accelerated evolutionary rate of *atpF* may enhance the efficiency of photosynthetic energy transduction systems. *clpP*, which encodes a proteolytic subunit of the Clp protease involved in importing proteins into plastids (Krause, 2012; Ravin et al., 2016), is presumed necessary for maintenance (Delannoy et al., 2011). A previous study has indicated that positive selection in the *clpP* gene is widespread in flowering plants (Erixon and Oxelman, 2008; Rockenbach et al., 2016; Williams et al., 2022). *matK* gene is suggested to be the plastid-encoded maturase of group II intron, and is therefore involved in the post-transcriptional processing of chloroplasts (Barthet and Hilu, 2007; Qu et al., 2018). In contrast to other groups of IIA ORFs, *matK* proteins lack domains that perform reverse transcriptase and endonuclease functions (Wicke et al., 2011). Positive selection sites in *matK* suggest positive selection fixes beneficial changes within East Asian lobelias. *psbK*, a subunit of photosystem II (Wu et al., 2020), was also positively selected. To our knowledge, the positive selection of *psbK* is uncommon in angiosperms. Positive selection may imply a unique ability of East Asian species to adapt to different light environments. *rbcL* is influenced by positive selection. This gene encodes the RuBisCO large subunit and plays a vital role in the adaptation of plants to environmental changes related to drought and low temperature. The positive selection of *rbcL* has also been described in some groups, such as *Brassicaceae* (Liu et al., 2012) and *IIex* (Aquifoliaceae) (Yao et al., 2019).

## 5 Conclusion

Early analyses used DNA fragmentation data to reconstruct the phylogenetic relationships of East Asian lobelias. Most current phylogenomic studies have suffered from the need to use more samples from the *L.* sect. *Hypsela*, *L.sect. Rhynchopetalum*, and *L.* sect. *Speirema.* Our phylogenomic framework inferred from complete plastomes illustrates the likely evolutionary relationships among East Asian lobelias based on all five representative sections. *L. melliana* was still found to be a sister species to the Oceanian and Afrotropic giant lobelias. Our study also demonstrates that the rarely used plastid marker in Lobelioideae phylogenetic reconstruction, *rpoC2*, shows considerable phylogenetic informativeness, which will advance the phylogenetic analyses of this subfamily. The IR/LSC boundaries, genome size, and the number of plastome repeats in *L.* sect. *Hypsela*, *L.* sect. *Speirema*, and *L.* sect. *Rhynchopetalum* were variable, indicating the non-conservative nature of the plastome structural evolution within these sections. In contrast, *L. zeylanica* and *L. heyne*ana had structural rearrangements in their LSC regions, suggesting they might have undergone different evolutionary histories. Identifying and authenticating species with medicinal significance are essential for the quality control of medicinal plants. We found highly divergent plastomes sequences, which would benefit future taxonomic work and population genetics studies of East Asian lobelias. Furthermore, the signatures of positive selection identified in some of the 74 protein-coding genes detected here provide insights into the role of plastomes in adaptive evolution. However, a more intensive sampling of Lobelioideae is essential for understanding their diversity. Moreover, the availability of plant plastomes will help elucidate the mode of molecular evolution.

## Data availability statement

The datasets presented in this study can be found in online repositories. The names of the repository/repositories and accession number(s) can be found below: Genbank accession numbers: OQ148738-OQ148751.

## Author contributions

C-JL collected samples, and wrote the manuscript; X-TX and H-XL collected samples and performed the analyses; R-NW performed the analyses; D-ZL designed the research and revised the manuscript. All authors contributed to the article and approved the submitted version.

## Supplementary material

Appendix S1 Repeats among the East Asian lobelias.
