# Supplementary figures and images for: Plastome evolution in the East Asian lobelias (Lobelioideae) using phylogenomic and comparative analyses
Source: Front Plant Sci. 2023 Mar 31;14:1144406. doi: 10.3389/fpls.2023.1144406 (PMC10102522; doi:10.3389/fpls.2023.1144406)

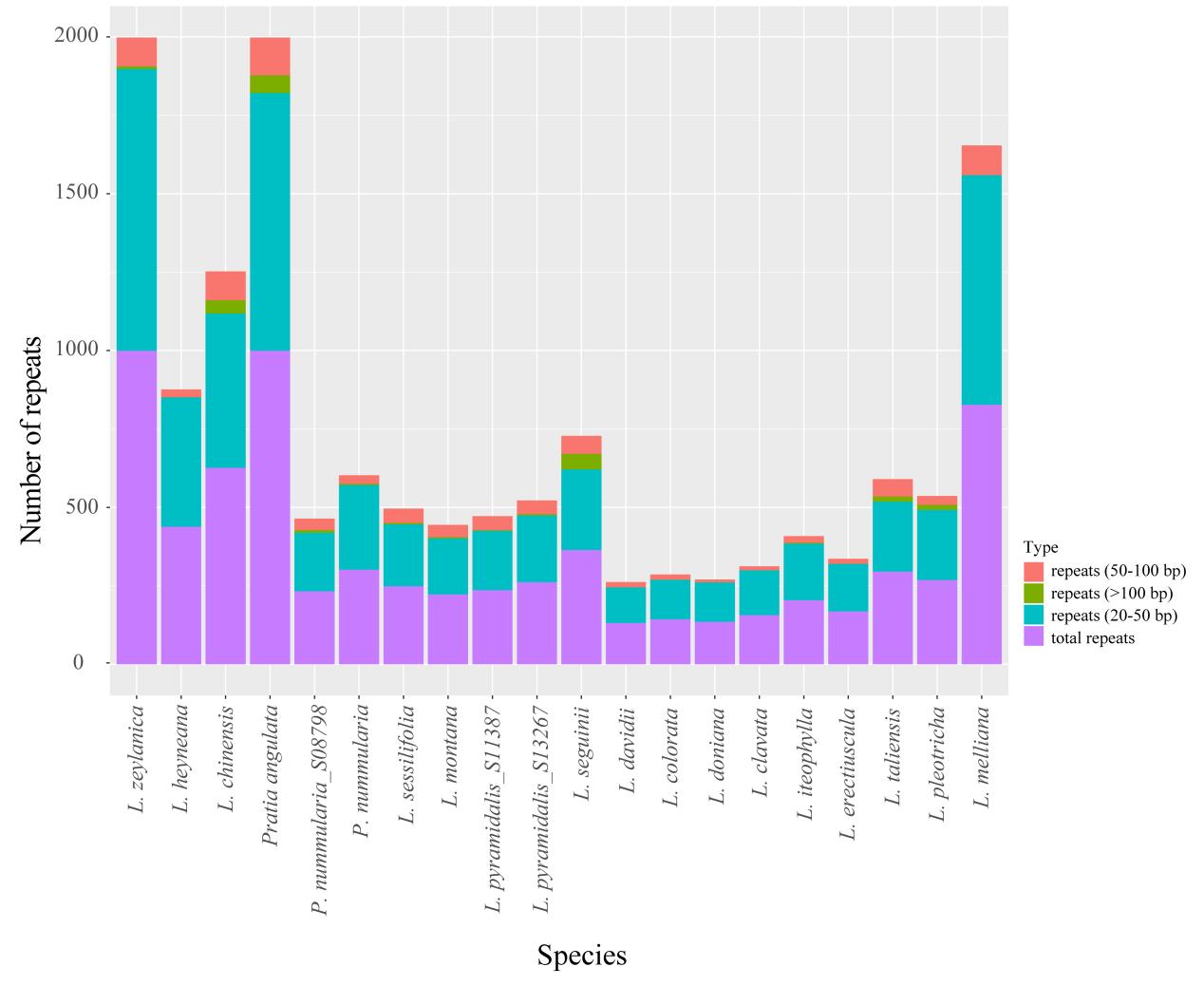

Supplement: Appendix S1 — Repeats among the East Asian lobelias. [file Image_1.jpeg]
