# Supplementary material for: Plastome evolution in the East Asian lobelias (Lobelioideae) using phylogenomic and comparative analyses
Source: Front Plant Sci. 2023 Mar 31;14:1144406. doi: 10.3389/fpls.2023.1144406 (PMC10102522; doi:10.3389/fpls.2023.1144406)
Supplement: Supplementary file 2 [file Table_1.docx]

**Table S1** Taxa included in the present study. NCBI accession numbers and voucher specimens’ information are provided for newly sequenced plastomes.

| **Family** | **Genus** | **Species** | **Sections of *Lobelia*** | **GenBank number**  **/voucher number** | **Collecting locations for newly sequenced species** |
| --- | --- | --- | --- | --- | --- |
| Lobeliaceae | *Lobelia* | *L. zeylanica*_S03968 | Section. *Delostemon* | OQ148751 | Hekou County, Honghe, Yunnan Province, China |
| Lobeliaceae | *Lobelia* | *L. zeylanica*_S11412 | Section. *Delostemon* | KUN1380246 | Pingbian Miao Autonomous County, Yunnan Province, China |
| Lobeliaceae | *Lobelia* | *L. alsinoides* | Section. *Delostemon* | KUN1380555 | Hongzhai Village, Gaoqiao Town, Lianjiang County, Zhejiang City, Guangdong Province, China |
| Lobeliaceae | *Lobelia* | *L. heyneana* | Section. *Stenotium* | OQ148743 | Eshan Yi Autonomous County, Yuxi City, Yunnan Province, China |
| Lobeliaceae | *Pratia* | *Pra. nummularia* | Section. *Hypsela* | OQ148746 | Yaorenshan National Forest Park, Sandu County, Qiannan, Guizhou Province, China |
| Lobeliaceae | *Lobelia* | *L. montana* | Section. *Rhynchopetalum* | OQ148744 | Malipo county, Wenshan, Yunnan Province, China |
| Lobeliaceae | *Lobelia* | *L. fangiana* | Section. *Rhynchopetalum* | KUN 173749 | [Mount](javascript:;) [Emei](javascript:;), Leshan City, Sichuan Province, China |
| Lobeliaceae | *Lobelia* | *L. pyramidalis*_S11387 | Section. *Rhynchopetalum* | OQ148748 | Xinping Yi and Dai Autonomous County, Yunnan Province, China |
| Lobeliaceae | *Lobelia* | *L. pyramidalis*_S13267 | Section. *Rhynchopetalum* | OQ148749 | Yuxi City, Yunnan Province, China |
| Lobeliaceae | *Lobelia* | *L. davidii* | Section. *Rhynchopetalum* | OQ148740 | Huaiping, Sanquan Village, Sanquan Town, Nanchuan District, Chongqing City, China |
| Lobeliaceae | *Lobelia* | *L. colorata* | Section. *Rhynchopetalum* | OQ148739 | Wenshan County, Yunnan Province, China |
| Lobeliaceae | *Lobelia* | *L. erectiuscula* | Section. *Rhynchopetalum* | OQ148742 | Motuo County, Tibet, China |
| Lobeliaceae | *Lobelia* | *L. taliensis* | Section. *Rhynchopetalum* | OQ148750 | Gongshan County, Yunnan Province, China |
| Lobeliaceae | *Lobelia* | *L. pleotricha* | Section. *Rhynchopetalum* | OQ148746 | Fugong County, Yunnan Province, China |
| Lobeliaceae | *Lobelia* | *L. doniana* | Section. *Rhynchopetalum* | OQ148741 | Diqing Tibetan Nationality, Yunnan Province, China |
| Lobeliaceae | *Lobelia* | *L. iteophylla* | Section. *Rhynchopetalum* | OQ148744 | Yongde County, Yunnan Province, China |
| Lobeliaceae | *Lobelia* | *L. clavata* | Section. *Rhynchopetalum* | OQ148738 | Jingdong Yi Nationality, Yunnan Province, China |
| Campanulaceae | *Trachelium* | *Trachelium caeruleum* |  | NC_010442 |  |
| Cyphiaceae | *Cyphia* | *Cyphia elata* var. *gerrardii* |  | MF770627 |  |
| Lobeliaceae | *Lobelia* | *L. heterophylla*subsp. *heterophylla* | *Lobelia* sect. *Holopogon* | MF770605 |  |
| Lobeliaceae | *Lobelia* | *L. physaloides* | Section. *Colensoa* | NC_036085 |  |
| Lobeliaceae | *Lobelia* | *L. baumannii* | Section. *Delostemon* | MF770633 |  |
| Lobeliaceae | *Lobelia* | *L. hartlaubii* | Section. *Delostemon* | MF770604 |  |
| Lobeliaceae | *Lobelia* | *L. holstii* | Section. *Delostemon* | MF770606 |  |
| Lobeliaceae | *Lobelia* | *L. malowensis* | Section. *Delostemon* | MF770609 |  |
| Lobeliaceae | *Lobelia* | *L. patula* | Section. *Delostemon* | MF770610 |  |
| Lobeliaceae | *Lobelia* | *L. thermalis* | Section. *Delostemon* | MF770614 |  |
| Lobeliaceae | *Lobelia* | *L. linearis* | Section. *Delostemon* | MF770608 |  |
| Lobeliaceae | *Lobelia* | *L. sonderiana* | Section. *Mezleriopsis* | MF770612 |  |
| Lobeliaceae | *Lobelia* | *L. galpinii* | Section. *Stenotium* | MF770603 |  |
| Lobeliaceae | *Lobelia* | *L. laxa* | Section. *Stenotium* | MF770607 |  |
| Lobeliaceae | *Lobelia* | *L. erinus* | Section. *Stenotium* | MF770635 |  |
| Lobeliaceae | *Lobelia* | *L. fervens* subsp. *fervens* | Section. *Stenotium* | MF770602 |  |
| Lobeliaceae | *Lobelia* | *L. anceps* | Section. *Stenotium* | KY354216 |  |
| Lobeliaceae | *Lobelia* | *L. jasionoides* | Section. *Jasionopsis* | KY354220 |  |
| Lobeliaceae | *Lobelia* | *L. polyphylla* | Section. *Tupa* | KY354224 |  |
| Lobeliaceae | *Lobelia* | *L. laxiflora* | Section. *Homochilus* | KY354221 |  |
| Lobeliaceae | *Lobelia* | *L. spicata* | Section. *Lobelia* | NC_036080 |  |
| Lobeliaceae | *Lobelia* | *L. siphilitica var. siphilitica* | Section. *Lobelia* | KY354225 |  |
| Lobeliaceae | *Lobelia* | *L. chinensis* | Section. *Hypsela* | MF061186 |  |
| Lobeliaceae | *Pratia* | *Pra. angulata* | Section. *Hypsela* | MF061180 |  |
| Lobeliaceae | *Pratia* | *Pra. nummularia* | Section. *Hypsela* | MF061203 |  |
| Lobeliaceae | *Lobelia* | *L. sessilifolia* | Section. *Rhynchopetalum* | MF061210 |  |
| Lobeliaceae | *Lobelia* | *L. melliana* | Section. *Rhynchopetalum* | MF061198 |  |
| Lobeliaceae | *Lobelia* | *L. yuccoides* | Section. *Revolutella* | MF061221 |  |
| Lobeliaceae | *Lobelia* | *L. niihauensis* | Section. *Revolutella* | MF061202 |  |
| Lobeliaceae | *Lobelia* | *L. kauaensis* | Section. *Tylomium* | MF061196 |  |
| Lobeliaceae | *Lobelia* | *L. mildbraedii* | Section. *Rhynchopetalum* | MF061199 |  |
| Lobeliaceae | *Lobelia* | *L. acrochila* | Section. *Rhynchopetalum* | MF061179 |  |
| Lobeliaceae | *Lobelia* | *L. rhynchopetalum* | Section. *Rhynchopetalum* | MF061206 |  |
| Lobeliaceae | *Lobelia* | *L. telekii* | Section. *Rhynchopetalum* | MF061216 |  |
| Lobeliaceae | *Lobelia* | *L. deckenii* subsp. *deckenii* | Section. *Rhynchopetalum* | MF061190 |  |
| Lobeliaceae | *Lobelia* | *L. burttii* subsp. *burttii* | Section. *Rhynchopetalum* | MF061183 |  |
| Lobeliaceae | *Lobelia* | *L. seguinii* | Section. *Rhynchopetalum* | MF061209 |  |
| Lobeliaceae | *Grammatotheca* | *G. bergiana* |  | MF770632 |  |
| Lobeliaceae | *Monopsis* | *M. alba* |  | MF770615 |  |
| Lobeliaceae | *Monopsis* | *M. stellarioides* subsp. *schimperiana* |  | MF770618 |  |
| Lobeliaceae | *Monopsis* | *M. debilis* var. *debilis* |  | MF770616 |  |
| Lobeliaceae | *Monopsis* | *M. flava* |  | MF770617 |  |
| Lobeliaceae | *Wimmerella* | *W. hederacea* |  | KY354229 |  |
| Lobeliaceae | *Centropogon* | *C. granulosus* |  | MF061166 |  |
| Lobeliaceae | *Burmeistera* | *B. auriculata* |  | NC_035778 |  |
| Lobeliaceae | *Burmeistera* | *B. borjensis* |  | NC_035777 |  |
| Lobeliaceae | *Siphocampylus* | *S. krauseanus* |  | KY045477 |  |
| Lobeliaceae | *Solenopsis* | *Sol. bivonae* |  | MF061224 |  |
| Lobeliaceae | *Palmerella* | *Pal. debilis* subsp. *serrata* |  | MF061222 |  |
| Lobeliaceae | *Legenere* | *Leg. valdiviana* |  | MF061177 |  |
| Lobeliaceae | *Porterella* | *Por. carnosula* |  | KY354228 |  |
| Lobeliaceae | *Downingia* | *D. cuspidata* |  | MF061171 |  |
| Lobeliaceae | *Downingia* | *D. elegans* |  | MF061172 |  |
| Lobeliaceae | *Diastatea* | *Dia. micrantha* |  | MF061170 |  |
| Lobeliaceae | *Hippobroma* | *H. longiflora* |  | MF061173 |  |
| Lobeliaceae | *Hypsela* | *Hyp. tridens* |  | MF061174 |  |
| Lobeliaceae | *Lithotoma* | *Lit. petraea* |  | KY354215 |  |
| Lobeliaceae | *Isotoma* | *I. hypocrateriformis* |  | NC_035363 |  |
| Lobeliaceae | *Dialypetalum* | *Dia. floribundum* |  | MF061169 |  |
| Lobeliaceae | *Apetahia* | *A. longistigmata* |  | MF061165 |  |
| Lobeliaceae | *Sclerotheca* | *Scl. viridiflora* |  | MF061223 |  |
| Lobeliaceae | *Cyanea* | *Cya. fissa* |  | KY354213 |  |
| Lobeliaceae | *Cyanea* | *Cya. leptostegia* |  | MF061168 |  |
| Lobeliaceae | *Clermontia* | *Cle. fauriei* |  | MF061167 |  |
| Lobeliaceae | *Brighamia* | *B. insignis* |  | NC_028633 |  |
| Lobeliaceae | *Delissea* | *Del. rhytidosperma* |  | KY354214 |  |
| Lobeliaceae | *Trematolobelia* | *T. kauaiensis* |  | MF061225 |  |
