# Supplementary material for: Plastome evolution in the East Asian lobelias (Lobelioideae) using phylogenomic and comparative analyses
Source: Front Plant Sci. 2023 Mar 31;14:1144406. doi: 10.3389/fpls.2023.1144406 (PMC10102522; doi:10.3389/fpls.2023.1144406)
Supplement: Supplementary file 3 [file Table_2.docx]

**Table S2** Primers used for assembly validation.

| **Primer** | **Sequence(5'>3')** | **Product size (bp)** |
| --- | --- | --- |
| LSC/IRa | GTTCGGTGCTAAGCCTCGGA ATCGGTCATGTTATGGTCAG | 521 |
| IRa/SSC | ACGAGTAACATGTACTTGCGGTGT CGGTGTAAGCGCTGGTAACA | 515 |
| SSC/IRb | GACAAGTCTGGTACACGCTC TGCCCTCACATGTGTGTTATTCT | 561 |
| IRb/LSC | GCTCAATAATCGCGGTGTAG CCGATGGCGTTATACCTAAC | 533 |

DNA amplification was performed for an initial 180s at 94 °C, followed by 35-40 cycles of 30s at 94 °C, 120s at 50 °C, 60s-90 at 72 °C and a final 420s at 72 °C. Reactions were carried out in a volume containing 2.0 mm/L MgCl_2_, 0.6 mm/L dNTP, 1μL of 10× buffer, 0.3 μmol/L of each primer, 1 U Taq DNA and 30 ng DNA template.
