# Supplementary material for: Plastome evolution in the East Asian lobelias (Lobelioideae) using phylogenomic and comparative analyses
Source: Front Plant Sci. 2023 Mar 31;14:1144406. doi: 10.3389/fpls.2023.1144406 (PMC10102522; doi:10.3389/fpls.2023.1144406)
Supplement: Supplementary file 5 [file Table_4.docx]

**Table S4** The best-fit evolutionary models of 22 partitioning subsets calculated by PartitionFinder. # Sites: the number of sites of the partitioning subsets.

| **Subset** | **Best Model** | **# Sites** | **Partition names** |
| --- | --- | --- | --- |
| 1 | GTR+I+G | 3573 | *ndhK, atpB, atpA* |
| 2 | GTR+I+G | 2175 | *ndhJ, psbH, atpE, psaI, petA* |
| 3 | GTR+G | 553 | *atpF, psbJ, psbA* |
| 4 | GTR+I+G | 2263 | *rbcL, psbT, atpH, petD, psbB, psbD, psbL, psbF, psbE* |
| 5 | GTR+I+G | 933 | *psbZ, atpI, rpl2, rpl16, rpl14* |
| 6 | GTR+I+G | 969 | *ccsA,* |
| 7 | GTR+I+G | 698 | *cemA, rps2, rpl22, rpl20, rpl32* |
| 8 | GTR+I+G | 574 | *clpP* |
| 9 | GTR+I+G | 1533 | *matK, rpoC1, rpoC2* |
| 10 | GTR+G | 1621 | *ndhG, ndhA* |
| 11 | GTR+I+G | 1531 | *ndhB, rps18, rps19, rps12* |
| 12 | GTR+I+G | 468 | *ndhC, psbM* |
| 13 | GTR+I+G | 1552 | *ndhD* |
| 14 | GTR+G | 496 | *rps15, rps16, ndhE, rpl33, rpl36* |
| 15 | GTR+I+G | 2230 | *ndhF, rps8* |
| 16 | GTR+I+G | 1188 | *ndhH, rps11, ndhI* |
| 17 | GTR+I+G | 504 | *ycf3, ycf4* |
| 18 | GTR+G | 2849 | *psaB, petB, rpoA, rpoB* |
| 19 | GTR+G | 578 | *psaJ, psbN, psbI, petG, petN* |
| 20 | GTR+I+G | 282 | *psbK, petL* |
| 21 | GTR+I+G | 3675 | *psbC, psaA* |
| 22 | GTR+I+G | 246 | *psaC, rps3, rps4, rps7* |
