# Supplementary material for: Plastome evolution in the East Asian lobelias (Lobelioideae) using phylogenomic and comparative analyses
Source: Front Plant Sci. 2023 Mar 31;14:1144406. doi: 10.3389/fpls.2023.1144406 (PMC10102522; doi:10.3389/fpls.2023.1144406)
Supplement: Supplementary file 6 [file Table_5.docx]

**Table S5** Phylogenetic informativeness profiles for 74 plastid protein-coding genes. Included are number of sites for which substitution rates were calculated (#Rates), net phylogenetic informativeness at epochs: 0-0.015 (Net PI) and per-site phylogenetic informativeness (Per-site PI). Ten genes with the most net informativeness are colored in red.

| **Loci** | **#Rates** | **Mean rate** | **SD** | **Net PI** | **Per-site PI** |
| --- | --- | --- | --- | --- | --- |
| *rpoC2* | 4763 | 103.7172574 | 870.0831587 | 88.48132227 | 0.934165128 |
| *rpoB* | 3216 | 1.170689739 | 2.144992183 | 59.07315096 | 0.536783276 |
| *ndhF* | 2235 | 5.327640671 | 158.6324569 | 37.77421735 | 0.166775919 |
| *matK* | 1525 | 1.881875148 | 2.533195064 | 33.06796857 | 0.282090103 |
| *psaA* | 2250 | 0.612913022 | 1.488340831 | 24.69811065 | 0.224379832 |
| *rpoC1* | 1314 | 18.36937983 | 358.0381082 | 23.75829693 | 0.212686133 |
| *psaB* | 2202 | 0.670992098 | 1.725983749 | 22.52235175 | 0.201120322 |
| *ndhD* | 1349 | 1.692867754 | 2.831255203 | 21.67782854 | 0.098016774 |
| *rpoA* | 1058 | 108.2289353 | 886.8691781 | 21.16436578 | 0.195692685 |
| *psbB* | 1524 | 0.823508202 | 1.873261033 | 18.00213769 | 0.162504808 |
| *atpA* | 1506 | 0.896588579 | 1.944592698 | 17.72182475 | 0.158839815 |
| *atpB* | 1497 | 0.879997729 | 1.987187155 | 17.64787103 | 0.161302085 |
| *ccsA* | 948 | 2.350299262 | 3.541357359 | 17.4350241 | 0.152292931 |
| *ndhB* | 1531 | 0.252516003 | 0.797443971 | 16.90116481 | 0.092638903 |
| *cemA* | 690 | 1.699411884 | 3.053139572 | 15.93880688 | 0.135693917 |
| *petA* | 963 | 1.015220042 | 1.915651806 | 15.06046793 | 0.135331946 |
| *rbcL* | 1425 | 0.89995186 | 2.495317821 | 14.75966637 | 0.133440618 |
| *rpl2* | 837 | 45.70643297 | 578.2225323 | 14.65870172 | 0.13878104 |
| *psbC* | 1420 | 0.625002606 | 1.589499323 | 14.00433828 | 0.12901382 |
| *rps3* | 762 | 72.11744987 | 715.7180801 | 13.35635831 | 0.127829245 |
| *rps2* | 718 | 32.94688482 | 484.0255171 | 13.33124866 | 0.120943434 |
| *atpF* | 552 | 1.173738949 | 1.867963943 | 13.27097188 | 0.126265177 |
| *ycf4* | 552 | 1.046178804 | 1.794122219 | 12.0004277 | 0.11356073 |
| *ndhK* | 681 | 1.022402056 | 1.907366415 | 11.14220956 | 0.049456197 |
| *rpl22* | 569 | 174.626164 | 1121.116385 | 10.64536598 | 0.091011195 |
| *rps4* | 636 | 60.68898286 | 662.7537304 | 10.49332603 | 0.093957832 |
| *psbA* | 1059 | 0.787413503 | 1.886544471 | 10.07023425 | 0.088560311 |
| *clpP* | 646 | 19.27900433 | 295.1716381 | 9.448088636 | 0.142335894 |
| *ndhH* | 1179 | 0.14911145 | 0.511974202 | 9.027072454 | 0.04562068 |
| *rps8* | 413 | 1.526359806 | 2.486937952 | 8.821111512 | 0.081051281 |
| *psbD* | 1059 | 0.579751747 | 1.516680127 | 8.748517451 | 0.078386591 |
| *rpl20* | 397 | 172.2813766 | 1117.439103 | 8.366814221 | 0.073397573 |
| *rpl16* | 399 | 1.478297995 | 2.462017815 | 8.273127751 | 0.076684574 |
| *atpI* | 741 | 0.891181781 | 2.029339295 | 8.062479331 | 0.07315585 |
| *ndhA* | 1098 | 0.201884062 | 0.840064493 | 8.006301602 | 0.042264747 |
| *rps11* | 414 | 2.790018841 | 4.124656418 | 7.412252607 | 0.063172572 |
| *rps7* | 468 | 1.073460684 | 2.883264174 | 7.312423802 | 0.072041086 |
| *ndhJ* | 474 | 0.858484177 | 1.662515156 | 7.13962263 | 0.031362459 |
| *atpE* | 399 | 1.120419549 | 2.159475937 | 6.873969539 | 0.064235051 |
| *ycf3* | 504 | 0.75008869 | 1.771514153 | 6.798076083 | 0.063028522 |
| *rpl14* | 366 | 1.265946175 | 2.417247055 | 6.584922848 | 0.063360557 |
| *rps18* | 510 | 136.0881729 | 988.0004119 | 6.386942141 | 0.071310126 |
| *rps15* | 327 | 1.070127217 | 1.971427035 | 6.052859016 | 0.0556749 |
| *ndhC* | 360 | 1.022015278 | 2.232170195 | 6.000270143 | 0.03132962 |
| *rps14* | 303 | 26.03380495 | 430.7966804 | 5.665319979 | 0.052746183 |
| *petB* | 645 | 0.656325736 | 1.689627152 | 5.285105026 | 0.045488408 |
| *rps16* | 270 | 30.30446852 | 456.2978611 | 4.896707717 | 0.040640997 |
| *rps19* | 279 | 1.969635125 | 3.187362012 | 4.688496765 | 0.042136541 |
| *ndhG* | 528 | 0.182489583 | 0.547757249 | 4.591626244 | 0.026139179 |
| *rpl32* | 203 | 668.1158335 | 2136.312865 | 4.162917349 | 0.039781599 |
| *ndhE* | 303 | 1.166484488 | 2.570215913 | 4.152935284 | 0.018392456 |
| *ndhI* | 489 | 0.195451943 | 0.629707691 | 4.103023359 | 0.019638239 |
| *rpl33* | 198 | 1.030655556 | 2.02538242 | 3.814288948 | 0.036776748 |
| *petD* | 417 | 0.841455156 | 2.27047379 | 3.800752036 | 0.033424243 |
| *psbH* | 219 | 0.766450685 | 1.578666379 | 3.623657971 | 0.03417461 |
| *psbK* | 183 | 1.122877596 | 1.962926926 | 3.477493314 | 0.031182014 |
| *psbZ* | 186 | 0.701756989 | 1.476396608 | 3.027808579 | 0.030167086 |
| *petN* | 87 | 1.229013793 | 1.715771832 | 2.822519262 | 0.027045675 |
| *psbE* | 249 | 0.472518876 | 1.421717934 | 2.654570234 | 0.024740702 |
| *atpH* | 243 | 0.748160905 | 1.936190926 | 2.434042604 | 0.023230613 |
| *psaJ* | 132 | 0.966372727 | 1.667534482 | 2.353521707 | 0.020013964 |
| *psaI* | 108 | 1.193231481 | 1.927081278 | 2.337553054 | 0.021941124 |
| *psbN* | 129 | 0.587271318 | 1.393840222 | 2.021339174 | 0.020421374 |
| *psaC* | 243 | 1.151114403 | 2.700445371 | 1.997647691 | 0.018109988 |
| *rpl36* | 111 | 1.277675676 | 2.287119996 | 1.988855768 | 0.018761692 |
| *psbI* | 108 | 0.859465741 | 1.699872279 | 1.955300628 | 0.018877628 |
| *petL* | 93 | 1.338065591 | 2.428458455 | 1.954017993 | 0.018183108 |
| *psbJ* | 120 | 0.84486 | 1.976237521 | 1.737941518 | 0.016972025 |
| *rps12* | 114 | 1.140570175 | 2.728641225 | 1.632177915 | 0.0139088 |
| *psbF* | 117 | 0.57134359 | 1.742721766 | 1.15047023 | 0.010816218 |
| *psbM* | 102 | 0.549898039 | 1.267223964 | 1.043342294 | 0.010148352 |
| *psbL* | 114 | 0.486421053 | 1.686368099 | 1.014887605 | 0.009211721 |
| *petG* | 111 | 0.572208108 | 1.538436839 | 0.768784728 | 0.006068099 |
| *psbT* | 99 | 1.029567677 | 2.41742968 | 0.672509573 | 0.005911895 |
